# Sir William Gowers on Premature Nervous Decay

**Published:** 1899-12-16

**Authors:** 


### SIR WILLIAM GOWERS ON PREMATURE NERVOUS DECAY.

At the last meeting of the Pathological Society Sir William Gowers described the view which he had long held as to the origin of various degenerative diseases of the nervous system, the essential secret of their mysterious incidence being that certain toxic agents so lower the durability of the nerve elements that some other factor, which might be harmless to uninjured nervous structures, induces in these enfeebled ones a premature decay. As he pointed out, an average man inherits an equal vitality in all his structures. They all decay together, so that " he goes to pieces at last like the famous ' one-hoss shay,' all at once, in mature, equable, senile decay." But there are individuals who possess certain structural elements (it may be in some part of their nervous or muscular system) so deficient in vital endurance that they decay as soon, or soon after, complete development is achieved. Yet these individuals live on, presenting symptoms which are due to the gradually extending decay of this or that portion of the nervous system. Among such cases are some of idiopathic muscular atrophy; cases of congenital atrophy of the optic nerve, in which sight fails in a whole family soon after puberty; and cases of " hereditary ataxy," or " Friedreich's disease," in which certain portions of the nervous system decay soon after adult age is reached. In other cases, again, the defect in vital endurance is only sufficient to render some structures less resistant than others, to cause, for instance, the motor nerve structures to decay before the others under the influence of grief, or the nutrition of the cerebral structures to give way under the strain of work or anxiety. Now, this disposition to decay, which is sometimes inherited in varying degrees, seems to be capable of being induced artificially, or at any rate as an accidental, non-hereditary tendency, by toxic and perhaps by other influences. The effect of such toxins is specific, and even though they may not continue to act for long, they produce an effect on nerve tissue which, under the strain and stress of other causes, may lead to failure of nutrition, decay, degeneration. When this degeneration shows itself it may appear to be spontaneous, or it may evidently have been excited; it may occur soon—that is, a few years after its cause has seemed active—or its effect may not become manifest until so long afterwards that the connection may only be perceptible, or indeed intelligible, by bearing in mind these considerations. Thus, although tabes may be a post-syphilitic disorder in middle life, it may also occur, while still in a true sense the result of syphilis, only when the general failure of approaching age obscures the significance of the cause—a cause which, indeed, did not directly produce the disease, but only led to that deficient durability of structure which made certain tissues decay out of their turn. " We shall understand many things more clearly," says Sir William Gowers, " if we perceive that acquired influences may have the effects of congenital dispositions, that either may cause effects unaided, and may predispose to the results of influences that would otherwise seem to act alone." In other words, it would seem that many degenerative diseases of the nervous system are due either to an inherent shortlivedness of nerve tissue or to a condition of enfeebled vitality which may make it easily succumb to stress and strain, and that enfeeblement may either be inherited or may be the result of exposure for a time to various toxic influences, one of which is that of syphilis.

